# Development of a Chemical Cocktail That Rescues Mouse Brain Demyelination in a Cuprizone-Induced Model

**DOI:** 10.3390/cells11071091

**Published:** 2022-03-24

**Authors:** Pei-Lun Lai, Chi-Hou Ng, Chia-Hsin Wu, Chien-Ying Lai, Scott C. Schuyler, Vicki Wang, Hsuan Lin, Yueh-Chang Lee, Ming-Hsi Chuang, Chang-Huan Yang, Wei-Ju Chen, Hsiao-Chun Huang, Jean Lu

**Affiliations:** 1 Genomics Research Center, Academia Sinica, Taipei 11529, Taiwan; coolman198411@yahoo.com.tw (P.-L.L.); littleleafmoon@gmail.com (C.-H.N.); chiahsin.ch.wu@gmail.com (C.-H.W.); believe614@hotmail.com (C.-Y.L.); josephyclee@mail.harvard.edu (Y.-C.L.); chloechen1026@gmail.com (W.-J.C.); 2 Genome and Systems Biology Degree Program, College of Life Science, National Taiwan University, Taipei 10617, Taiwan; 3 Department of Life Science, Institute of Molecular and Cellular Biology, College of Life Science, National Taiwan University, Taipei 10617, Taiwan; 4 Department of Biomedical Sciences, College of Medicine, Chang Gung University, Taoyuan 33302, Taiwan; schuyler@mail.cgu.edu.tw; 5 Division of Head and Neck Surgery, Department of Otolaryngology, Chang Gung Memorial Hospital, Taoyuan 33302, Taiwan; 6 Translational Medicine, National Defense Medical Center, Academia Sinica, Taipei 11490, Taiwan; y103110@gmail.com; 7 Taiwan International Graduate Program in Molecular Medicine, National Yang Ming Chiao Tung University, Academia Sinica, Taipei 11221, Taiwan; johnnylin0917@gm.ym.edu.tw; 8 Department of Ophthalmology, Hualien Tzu Chi Hospital, Buddhist Tzu Chi Medical Foundation, Hualien 97002, Taiwan; 9 College of Management, Chung Hua University, Hsinchu 30012, Taiwan; mercy@gwoxi.com; 10 Gwo Xi Stem Cell Applied Technology Co., Ltd., Hsinchu 30261, Taiwan; at60279@hotmail.com; 11 Graduate Institute of Electronics Engineering, College of Electrical Engineering and Computer Science, National Taiwan University, Taipei 10617, Taiwan; 12 National Core Facility Program for Biotechnology, National RNAi Platform, Academia Sinica, Taipei 11529, Taiwan; 13 Department of Life Science, Tzu Chi University, Hualien 97004, Taiwan; 14 Graduate Institute of Medical Sciences, National Defense Medical Center, Taipei 11490, Taiwan

**Keywords:** oligodendrocytes, oligodendrocyte progenitor cells (OPCs), multiple sclerosis (MS), induced oligodendrocyte-like cells (iOLCs), cuprizone-induced model, demyelination, remyelination

## Abstract

Oligodendrocytes are glial cells located in the central nervous system (CNS) that play essential roles in the transmission of nerve signals and in the neuroprotection of myelinated neurons. The dysfunction or loss of oligodendrocytes leads to demyelinating diseases such as multiple sclerosis (MS). To treat demyelinating diseases, the development of a therapy that promotes remyelination is required. In the present study, we established an in vitro method to convert human fibroblasts into induced oligodendrocyte-like cells (iOLCs) in 3 days. The induced cells displayed morphologies and molecular signatures similar to oligodendrocytes after treatment with valproic acid and exposure to the small molecules Y27632, SU9516, and forskolin (FSK). To pursue the development of a cell-free remyelination therapy in vivo, we used a cuprizone-induced demyelinated mouse model. The small molecules (Y27632, SU9516, and FSK) were directly injected into the demyelinated corpus callosum of the mouse brain. This combination of small molecules rescued the demyelination phenotype within two weeks as observed by light and electron microscopy. These results provide a foundation for exploring the development of a treatment for demyelinating diseases via regenerative medicine.

## 1. Introduction

Oligodendrocytes, also known as oligodendroglia, are a type of neuroglia that generate myelin sheaths that wrap around axons in the CNS to create an electrical insulation that increases the rate of electrical conduction [1,2]. Generally, one oligodendrocyte wraps around approximately 50 axons and, in myelinated neurons, increases the travel rate of nerve impulses to approximately 100 times faster that of non-myelinated neurons [3]. In addition, oligodendrocytes help nerve regeneration by providing nutrients and an appropriate environment [4,5,6,7]. During development, oligodendrocytes are derived from oligodendrocyte progenitor cells (OPCs), which are distributed in the central nervous system and repair demyelinated axons to form new myelin sheaths [8,9]. Therefore, promoting the recruitment or differentiation of OPCs is considered to be the main strategy for enhancing myelination and developing novel treatments against demyelinating diseases [10].

Myelin loss and dysfunction affects approximately 2–2.5 million people worldwide [11]. Multiple sclerosis (MS) is one of the most common chronic demyelinating diseases caused by the loss of oligodendrocytes when the immune system attacks the myelin sheath [12,13,14]. Damaged demyelinated areas in the CNS contribute to the development of MS symptoms including difficulties in or damage to vision, bladder, memory and thinking, pain, cramps, speech, and swallowing. MS can also cause stroke, muscle weakness, or death [15]. Several studies in which OPCs were injected into damaged areas to ensheath the demyelinated region yielded promising results [16,17,18]. However, it is always hard to obtain sufficient human primary OPCs for cell therapy [16,17,18]. Therefore, producing enough OPCs and promoting remyelination by glial cells is a key issue of concern. Currently, human embryonic stem cells (hESCs) and induced pluripotent stem cells (iPSCs) provide a source material for the production of OPCs through the ectopic expression of transcription factors accompanied by soluble factors to promote differentiation [19,20,21]. Studies have shown that both neural stem cells (NSCs) derived from hESCs and OPCs derived from mesenchymal stem cells (MSCs) improve the symptoms of myelin damage in animal experiments [22,23,24]. However, OPCs produced by viral transduction have a higher risk of insertion mutations, and the differentiation process of ESCs and iPSCs is laborious and takes approximately 60–100 days; the process of differentiation is also costly and involves the risk of teratoma formation [25,26,27,28,29]. Therefore, the use of small molecules to generate induced oligodendrocyte-like cells (iOLCs) is an attractive method to pursue.

Liu et al. used a chemical approach to convert mouse fibroblasts into induced OPCs for cell therapy [30]. Tsui et al. used induced OPCs from rat MSCs as cell material to treat demyelination [24]. However, cell therapy still suffers from issues such as immune rejection and low rates of survival of transplanted cells in vivo, so cell-free therapy is still an attractive alternative treatment strategy compared to cell-based therapy [31]. Chen et al. studied the impairment of motor function in a rat model of Parkinson’s disease [32] using factors isolated from stem-cell-conditioned medium (CM). Numerous studies have also shown related cell-free approaches for the treatment of neurological diseases under various experimental conditions [31]. Some studies have also directly injected drugs into demyelinated models to observe the state of repair [33,34,35,36]. Therefore, cell-free therapy for demyelination using drugs discovered in cell reprogramming research is feasible and promising.

Cuprizone causes demyelination of the cerebrum, cerebellar nuclei, brainstem, and the corpus callosum (CC) and can be used to study different aspects of MS pathology in mice models [37,38]. In this study, we generated iOLCs from human fibroblasts after treatment with three major small molecules, i.e., Forskolin, Y27632, and Su9516, after valproic acid (VPA) pre-treatment, and in three days, these cells expressed multiple oligodendrocyte markers. We then investigated the remyelination potential of this chemical combination by injecting these three small molecules directly into the corpus callosum of a cuprizone-induced demyelinated mouse brain model. We observed that our compound formula promoted remyelination in vivo. Our work showed that small molecules can be directly injected into areas of the brain where myelin has been damaged, and that this approach is convenient and promising for treating demyelinating diseases.

## 2. Materials and Methods

### 2.1. Cell Lines and Culture Condition

Human normal foreskin skin fibroblast primary cells (CRL-2097) were obtained from the ATCC. CRL-2097 cells were cultured at 37 °C with 5% CO_2_ in high-glucose Dulbecco’s Modified Eagle’s Medium (HG-DMEM) (Life Technologies, Carlsbad, CA, USA) supplemented with 10% fetal bovine serum (FBS) (GE Healthcare, Chicago, IL, USA). Cell viability was determined by trypan blue staining. All of the cultures were free of mycoplasma. This study was approved by the Academia Sinica Ethics Committee (IRB number: AS-IRB-BM-17008, AS-IRB-BM-20038).

### 2.2. Cell Trans-Differentiation

Cells (1 × 10^4^ per well) were plated on 24-well plates. Cells were cultured in HG-DMEM (Life Technologies, Carlsbad, CA, USA) supplemented with 10% fetal bovine serum (FBS) (Hyclone) for 2 days. Then, the medium was replaced with DMEM/F12 (Thermo Fisher, Waltham, MA, USA) with 1% N2 supplement, 1% B27 supplement (both from Life Technologies, Carlsbad, CA, USA), 20 ng/mL platelet-derived growth factor AA (PDGF-AA), 20 ng/mL epidermal growth factor (EGF), 20 ng/mL basic fibroblast growth factor (bFGF), 20 ng/mL neurotrophin-3 (NT3) (all from Peprotech, Cranbury, NJ, USA). Then, the cells were treated with 3 mM valproic acid (Tocris) for 2 days and 10 µM Y27632 (LC Laboratories, Woburn, MA, USA), 10 µM SU9516 (R & D Systems, Minneapolis, MN, USA), and 10 µM forskolin (FSK) (Tocris, Bristol, UK) for 1 day. The reagent is listed in Supplementary Tables S1 and S2.

### 2.3. Immunofluorescence Assay

Cells were fixed with 4% formaldehyde for 15 min at room temperature and washed once with 1 × phosphate-buffered saline (PBS). Cells were permeabilized with 0.3% Triton X-100 for 5 min, washed twice with 1 × PBS, and blocked in 2% bovine serum albumin (BSA) in PBS for 30 min. Then, the cells were incubated with an oligodendrocyte-specific marker 4 (O4) antibody (Merck Millipore, Burlington, MA, USA), oligodendrocyte transcription factor 2 (Olig2) antibody (Merck Millipore, Burlington, MA, USA), galactosylceramidase (GalC) antibody (Proteintech, St. Leon-Rot, Germany), myelin proteolipid protein (PLP) antibody (Abcam, Cambridge, UK), myelin basic protein (MBP) antibody (Proteintech, Sankt Leon-Rot, Germany), and 4′,6-diamidino-2-phenylindole (DAPI) (Life Technologies, Carlsbad, CA, USA) in blocking buffer (2% BSA in PBS) overnight at 4 °C. The detailed information of the antibodies is listed in Supplementary Table S3. Cells were washed twice in 1 × PBS, followed by incubation in CF555 goat anti-rabbit secondary antibody (Life Technologies, Carlsbad, CA, USA), CF488 goat anti-rabbit secondary antibody (Biotium, Fremont, CA, USA), CF555 goat anti-mouse secondary antibody (Life Technologies, Carlsbad, CA, USA), CF488 goat anti-mouse secondary antibody (Biotium, Fremont, CA, USA), or CF555 goat anti-rat secondary antibody (Life Technologies, Carlsbad, CA, USA) in blocking buffer for 1 h in the dark at room temperature. Cells were washed twice in 1 × PBS. Slides were mounted with ProLong Gold anti-fade reagents (Thermo Fisher, Waltham, MA, USA). Fluorescence was analyzed using a fluorescence microscope (Axiovert 200 M, Zeiss, Oberkochen, Germany).

### 2.4. Real-Time Quantitative PCR Assays

RNA was isolated from cells using the RNeasy Micro Kit (Qiagen, Germantown, MD, USA) and was reverse-transcribed (400 ng). RT-PCR analysis was performed with KAPA SYBR FAST qPCR Kits (Kapa Biosystems, Wilmington, MA, USA), and the housekeeping gene, Glyceraldehyde-3-Phosphate Dehydrogenase (GAPDH), was used for the endogenous reference. The primers are listed in Supplementary Table S4.

### 2.5. Flow Cytometry

Cells were harvested into 1.5 mL tubes filled with Accutase, washed 3 times with cold PBS, blocked with 2% BSA in PBS for 15 min, and then stained with fluorescent-conjugated antibody A2B5 (Miltenyi Biotec, Bergisch Gladbach, North Rhine-Westphalia, Germany) for 30 min. After 3 washes with PBS, cells were transferred to FACS buffer. A BD FACSCanto II (Franklin Lakes, NJ, USA) flow cytometer was used for the quantification of A2B5+ cells.

### 2.6. Western Blot Analysis

Cells were collected by scraping in lysis buffer (1% NP40, 50 mM Tris pH 8.0, 150 mM NaCl, 2 mM EDTA, 1 mM Na_3_VO_4_) and protease inhibitor cocktail (Sigma, Burlington, MA, USA). Protein quantification was performed with a Bio-Rad protein assay (Bio-Rad, Hercules, CA, United States). Samples were boiled for 15 min at 100 °C, 30 µg protein was loaded on 10% acrylamide gels, and electrophoresis was started at 90 V for 15 min, then turned to 120 V for 1 h. Amersham Protran 0.45 µm nitrocellulose membranes were used for blotting. Blots were blocked for 30 min by shaking in 5% BSA in phosphate-buffered saline with Tween 20 (PBST)(Sigma, Burlington, MA, USA). Primary antibodies (NK2 Homeobox 2 (Nkx2.2), Proteintech, Sankt Leon-Rot, Germany) were diluted in 5% BSA in PBST and incubated overnight at 4 °C on the shaker. Secondary antibodies were diluted in 5% BSA in PBST and cultured for 1 h at room temperature on the shaker. Detection of protein signals was performed with the UVP BioSpectrumAC system (Jena, Thuringia, Germany).

### 2.7. Cuprizone-Induced Mouse Model Treated with Chemicals

We used 8–10-week-old C57/BL6 mice for the cuprizone-induced-demyelination animal model. The mice were divided into three groups. The first group was fed a normal diet, which served as the positive control. For the two other groups, food was mixed with 0.2% (*w*/*w*) cuprizone as the diet. After 12 weeks of the cuprizone diet, mice were randomized into 2 groups. One group was injected with the chemical cocktail, and the second group was injected with PBS. For the chemical and PBS injections, Zoletil and Ropum (1:1) were used for anesthesia, and the injection site was determined by a stereotaxic apparatus. Mice received 4 µL of the chemical mix (100 µM of Y27632, SU9516, and forskolin) and were subjected to a 10 min deposition time at 0.8 µL/min at the following coordinates: Bregma: +0.98 mm (antero-posterior axis), −1.75 mm (outer axis), −2.25 mm (vertical axis). One group received a 4 µL PBS injection as a control. At 14 days post-injection, transcardiac perfusion was prepared by pre-rinsing the tissue with 2% paraformaldehyde (PFA) in PBS, pH 7.4. Brains were explanted and then sectioned; immunohistochemical and electron microscopy analyses of the corpus callosum were performed (see below). The animal experiments were approved by the Academia Sinica Committee (IACUC number: 18-02-1191).

### 2.8. Luxol Fast Blue

Corpus callosum slices were prepared by transcardial perfusion and embedded in epoxy resin embedding medium (Sigma, Burlington, MA, USA). A microtome was used to cut the corpus callosum into 7 μm thick sections. Sections were stained with Luxol fast blue (LFB) solution. The area of Luxol fast blue was quantified by ImageJ analysis software.

### 2.9. Electron Microscopy

For electron microscopy analysis, transcardial perfusion was performed with fixative (4% paraformaldehyde and 2.5% glutaraldehyde in PBS, pH 7.4). The brains were explanted and kept overnight at 4 °C in fixative. The brains were cut into 1 mm slices and kept in fixative. The corpus callosum was cut into 1 mm × 1 mm × 2 mm tissue blocks and post-fixed in 1% osmium tetroxide (*w*/*v*). The blocks were dehydrated and embedded in Spurr resin (Spurr Low Viscosity Embedding Kit; Electron Microscopy Sciences (EMS), Hatfield, PA, USA). After examining 0.5 µm semi-thin sections with toluidine blue staining for the presence of relevant regions, ultra-thin sections (60 nm) were taken and counter-stained with uranyl acetate and lead citrate and examined using an FEI Tecnai G2 F20 S-TWIN transmission electron microscope (Hillsboro, OR, USA).

### 2.10. Statistical Analysis

All statistical data were presented as the mean ± standard deviation (S.D.) of at least three biological replicates. Statistical analysis was performed by un-paired, two-tailed *t*-tests and one-way ANOVA with GraphPad Prism 8.2.1 (GraphPad Software, San Diego, CA, USA), where a *p*-value < 0.05 was considered a significant difference. With ordinary one-way ANOVA, the post hoc multiple comparisons tests were Tukey’s multiple comparisons test.

## 3. Results

### 3.1. Chemical Reprogramming of Fibroblasts into an Oligodendrocyte-Like State In Vitro

**Figure 1:**
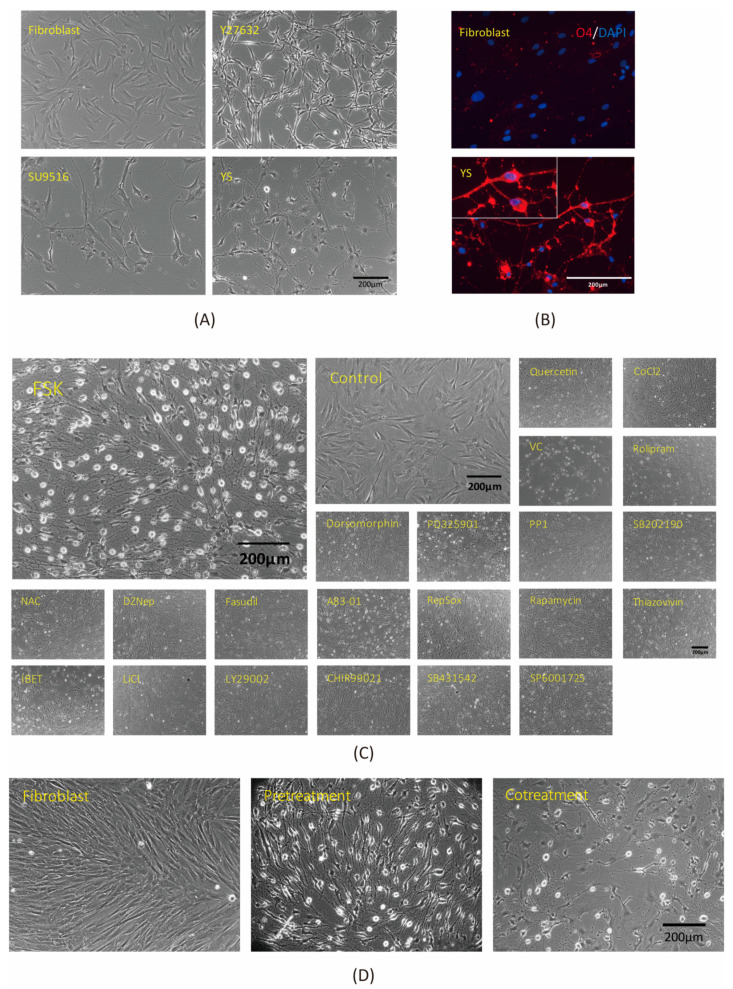
High-throughput screening of small molecules for conversion of fibroblasts into oligodendrocyte-like cells. (**A**) Fibroblasts changed cell morphology after treatment with different chemicals. Phase-contrast images represent solvent control (fibroblasts) (top left), Y27632-treated fibroblasts (top right), SU9516-treated fibroblasts (bottom left), and fibroblasts treated with a combination of Y27632 and SU9516 (bottom right). (**B**) Immunofluorescence staining showed the oligodendrocyte-specific marker O4 on induced cells (red). DAPI (blue) was used to stain nuclei. (**C**) We tested 1540 chemicals, and the candidate molecules that promoted reprogramming were screened on the basis of Y27632 and SU9516 after valproic acid (VPA) treatment. These images show different chemical combination treatments of fibroblasts. Forskolin (FSK)-treated cells maintained dome-shaped cell morphology and dendritic structures. The ingredients of ‘VYSF’ included valproic acid, Y27632, SU9516, and forskolin. (**D**) Testing of VPA. Pre-treated and co-treated VPA with ‘YSF’ cocktails. Fibroblasts were treated with VPA for 2 days and then treated with the YSF mixture for 1 day or fibroblasts were co-treated with VPA and YSF mixture for 1 day. Phase-contrast images representative of solvent control (fibroblasts) (left), VPA pre-treatment (middle), and VPA combination treatment (right) of cells are shown. VYSF refers to valproic acid, Y27632, SU9516, and forskolin, respectively.

To find out which chemicals could convert fibroblasts into oligodendrocyte-like cells, we screened chemically treated cells on the basis of morphological changes that included the formation of a dendritic morphology of neural lineage cells and the dome-shaped morphology of the cell bodies of oligodendrocytes. In a preliminary screen, two chemicals, Y27632 and SU9516, were identified that may have been involved in triggering the generation of oligodendrocyte-like cells from human primary fibroblasts (CRL2097). We observed that Y27632 and SU9516 treatment induced morphological changes in fibroblasts, which became dendritic and dome-shaped (Figure 1A). Therefore, we combined Y27632 and SU9516 into a cocktail to convert fibroblasts into an oligodendrocyte-like morphology (Figure 1A). To characterize the induced oligodendrocyte-like cells, we performed immunofluorescence staining to analyze the expression of oligodendrocyte-specific markers. We initially analyzed the expression of oligodendrocyte-specific marker 4 (O4) and found that the Y27632- and SU9516-treated groups had higher O4 expression compared with the solvent control group (Figure 1B).

Before the next screening step, we noted that valproic acid (VPA), which is a histone deacetylase (HDAC) inhibitor commonly used in different reprogramming protocols, had been suggested to enhance reprogramming by modifying histones or other pathways in human and mouse fibroblasts [39,40,41]. Therefore, we added an initial treatment with VPA as a two-step treatment to facilitate the transformation of fibroblasts into oligodendrocyte-like cells. VPA has also been reported to enhance the chemical reprogramming of Schwann cells and neural-precursor-like cells [42,43].

**Figure 2:**
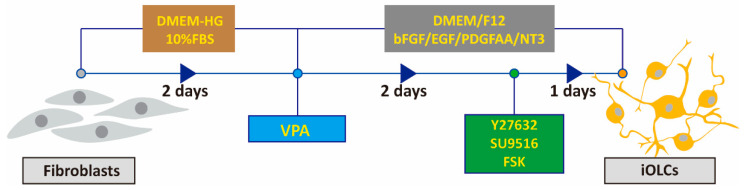
Sketch of iOLCs generation process. The flow chart shows Y27632, SU9516, and FSK treatment with induction medium one day after VPA pre-treatment and after two days of treatment that converted fibroblasts into iOLCs.

Next, to optimize the two-chemical cocktail, we performed an additional high-throughput screen using a chemical library of over 1500 molecules purchased from Selleckchem. The chemicals were rescreened for the ability to induce morphological changes in fibroblasts on top of a three-chemical formula including valproic acid (VPA), Y27632, and SU9516. Forskolin (FSK) was selected for its ability to induce morphological changes, and most cells exhibited prominent oligodendrocyte-like morphology (Figure 1C). FSK is an activator of cyclic adenosine monophosphate (cAMP), which induces rat OPC maturation by up-regulating oligodendrocyte-specific proteins such as 2′,3′-Cyclic-nucleotide 3’-phosphodiesterase (CNP), myelin oligodendrocyte glycoprotein (MOG), and myelin proteolipid protein (PLP) [44]. We found that FSK increased the network complexity of oligodendrocyte-like cells under the combined action of VPA, Y27632, and SU9516. Cells were treated with VPA for two days, followed by treatment with Y27632, SU9516, and FSK for one day. We investigated the co-processing of VPA with the three other chemicals in the cocktail; however, our experiments showed that only the pre-treatment improved the conversion efficiency (Figure 1D). For cell transdifferentiation, our final optimized cocktail transformed cells into an oligodendrocyte-like state, in terms of morphology. We found that the four chemicals in combination efficiently converted fibroblasts to oligodendrocyte-like cells within three days (Figure 2).

### 3.2. Characterization of Chemically Induced Oligodendrocyte-Like Cells

**Figure 3:**
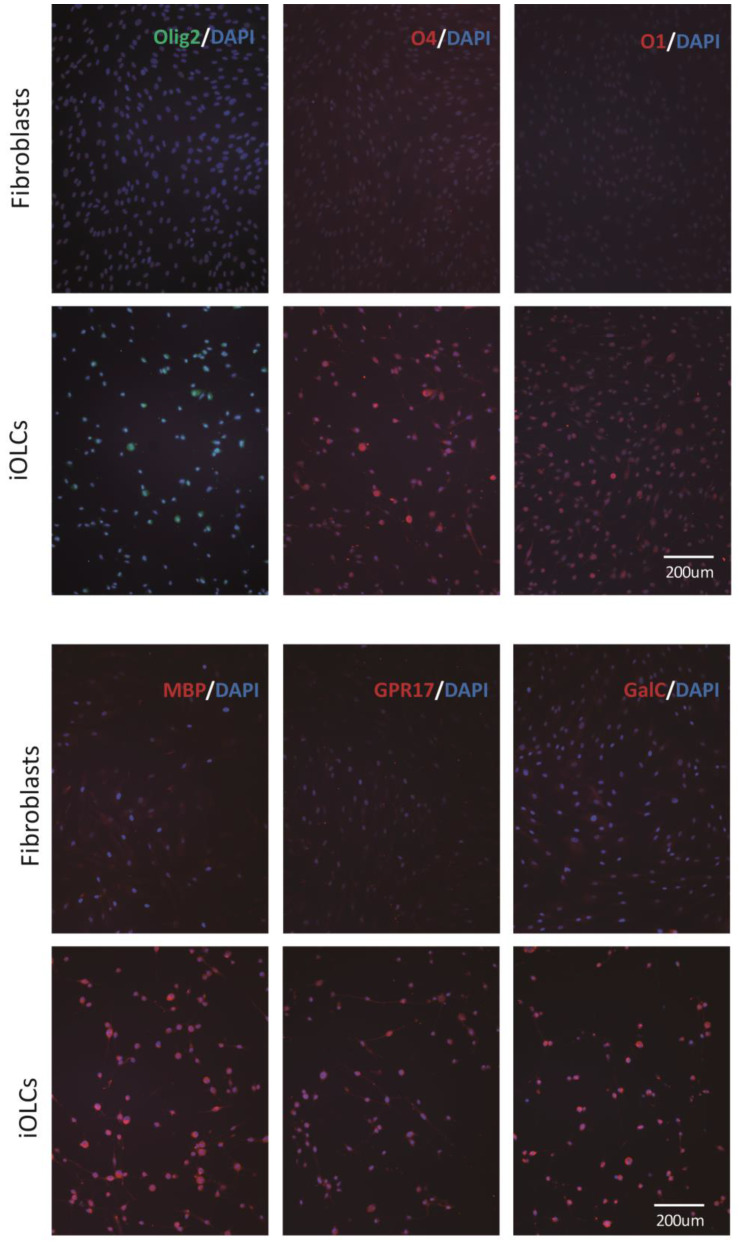
Expression of oligodendrocyte markers observed by immunofluorescence on iOLCs induced with four chemicals. iOLCs induced by the VYSF cocktail are compared with the solvent control (fibroblasts) group. To start, 1 × 10^4^ fibroblasts were seeded in each well of 24-well plates; after two days of expansion, fibroblasts were treated with the VYSF cocktail. Data showing protein expression, including oligodendrocyte-specific transcription factor Olig2 (upper left, green); oligodendrocyte-specific surface antigens O4 (upper–middle, red) and O1 (upper right, red); and oligodendrocyte-specific markers MBP (bottom left, red), GPR17 (bottom–middle, red), and galactosylceramidase (bottom right, red). DAPI (blue) was used to stain nuclei. VYSF refers to valproic acid, Y27632, SU9516, and forskolin, respectively.

**Figure 4:**
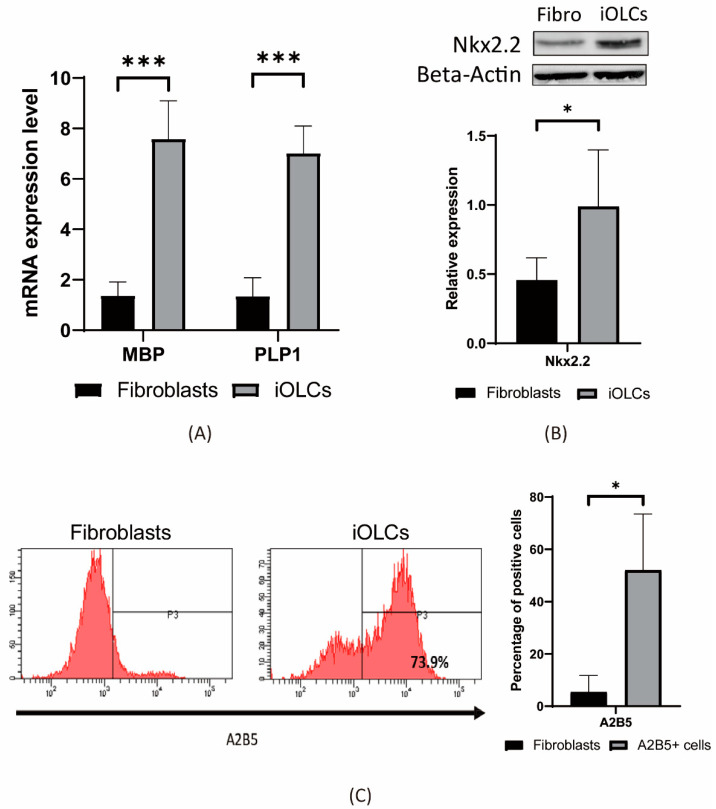
Marker expression in chemically induced oligodendrocyte-like cells. iOLCs were the cells treated with valproic acid, Y27632, SU9516, and forskolin (VYSF). (**A**) The mRNA expression of myelin-forming proteins MBP and PLP1 between the VYSF-treated group (iOLCs) and the solvent control group was confirmed by quantitative real-time polymerase chain reaction (qRT-PCR). Data are presented as mean ± standard deviation. (*n* = 3). GAPDH was used as reference gene. Significant differences were determined using Student’s *t*-test, * *p* < 0.05, ** *p* < 0.005, *** *p* < 0.001. (**B**) Western blot analysis showing transcription factor Nkx2.2 protein up-regulation in the VYSF-treated group (iOLCs). Quantification of Western blot data is shown at the bottom. Data are presented as mean ± standard deviation. (*n* = 5). β-actin was used as an internal control. Significant differences were determined using Student’s *t*-test, * *p* < 0.05. (**C**) Flow cytometry was performed to quantify A2B5+ cells of chemical cocktail VYSF-treated fibroblasts (iOLCs). In the chemically treated group, A2B5+ was detectable in an average of 50% of the cells. Data are presented as mean ± standard deviation. (*n* = 3). Significant differences were determined using Student’s *t*-test, * *p* < 0.05.

To characterize the induced cells, we examined expression of markers in the iOLCs using immunofluorescence, quantitative reverse transcription polymerase chain reaction (qRT-PCR), flow cytometry, and Western blotting. For the immunofluorescence experiments, we stained for the oligodendrocyte markers Olig2, Oligodendrocyte-specific marker 1 (O1), O4, Galactosylceramidase (GalC), G-protein-coupled receptor 17 (GPR17), and myelin basic protein (MBP). O1, O4, Olig2, GPR17, GalC, and MBP were all expressed in the chemical-cocktail-treated cells (Figure 3). In addition, we found the expression levels of PLP1 and MBP were increased by qRT-PCR (Figure 4A). The transcription factor, NK2 Homeobox 2 (Nkx2.2), associated with OPC differentiation, was up-regulated by the Western blot analysis (Figure 4B). The results showed that Nkx2.2, Proteolipid protein 1 (PLP1), and MBP were all highly expressed. To quantify the induced oligodendrocyte-like cells, we chose the surface antigen, A2B5, of oligodendrocyte progenitor cells as the basis for quantification by flow cytometry, and the data indicated that approximately 50% of these cells were positive (Figure 4C). Taken together, our data suggested that most fibroblasts were directed to transdifferentiate into oligodendrocyte lineage cells.

### 3.3. Direct Chemical Injection Ameliorates Cuprizone-Induced Demyelination in Mice

**Figure 5:**
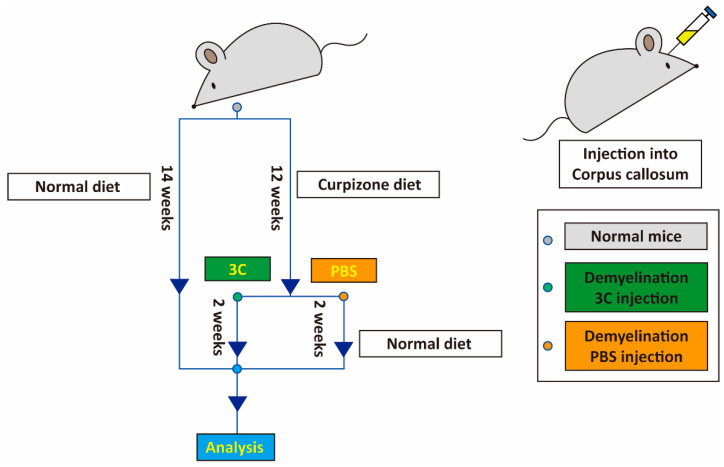
Sketch of remyelination improvement with injected 3-chemical (3C) cocktail into a cuprizone-induced-demyelination mouse model. The flow chart shows that the 3C cocktail (Y27632, SU9516, and FSK) and PBS were injected into mice in the corpus callosum after 12 weeks of cuprizone-induced demyelination. After 2 weeks of recovery, 3C cocktail (Y27632, SU9516, and FSK) injection groups, PBS injection groups, and positive control groups (normal diet) were sacrificed for the follow-up analysis.

**Figure 6:**
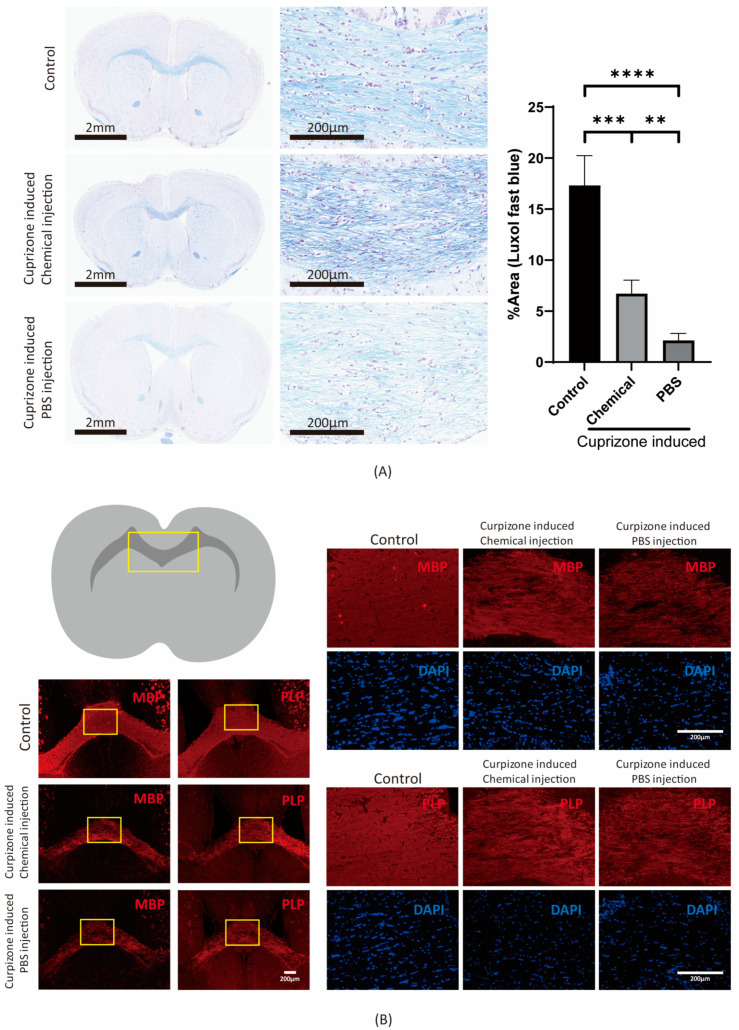
Chemical cocktail injection rescues cuprizone-induced demyelination in mice. Induction of demyelination in B6 mice by cuprizone. The YSF cocktail and PBS were injected directly into the corpus callosum of mice; then, the mice were allowed to recover for two weeks before dissection. YSF refers to Y27632, SU9516, and forskolin. (**A**) Luxol fast blue staining was performed to detect the remyelination process in the corpus callosum. The quantification of Luxol fast blue was compared between the YSF-cocktail- and PBS-injected groups after 2 weeks of recovery before dissection. Data are presented as mean ± standard deviation. (*n* = 6). Significant differences were determined by ANOVA followed by Sidak’s multiple comparisons test, * *p* < 0.05, ** *p* < 0.005, *** *p* < 0.001, **** *p* < 0.0001. (**B**) After 2 weeks of recovery, immunofluorescence staining was performed to observe the protein expression of the YSF-cocktail- and PBS-injected groups. Comparison of myelination densities for MBP (top) and PLP (bottom) protein expression in the corpus callosum of the YSF-cocktail- and PBS-injected groups. DAPI (blue) was used to stain nuclei.

In our experiments, a chemical cocktail induced the formation of oligodendrocyte-like cells in vitro (Figure 1, Figure 2, Figure 3 and Figure 4). To test the ability of the cocktail mixture to promote remyelination in vivo, we hypothesized that the chemical cocktail would directly promote remyelination in the corpus callosum. A murine model of cuprizone-induced demyelination was used to study the effects of the chemotherapy cocktail (Figure 5). After 12 weeks of diet-induced demyelination by cuprizone, the three-chemical (3C) cocktail (Y27632, FSK, and SU9516) and a PBS control, were injected directly into the mouse corpus callosum. After 2 weeks of recovery, Luxol fast blue staining revealed increased levels of remyelination in the chemical cocktail injection group as compared with the PBS control group (Figure 6A). Likewise, in immunofluorescence staining, the protein levels of MBP and PLP were higher in the chemical injection group compared with the PBS control group (Figure 6B).

**Figure 7:**
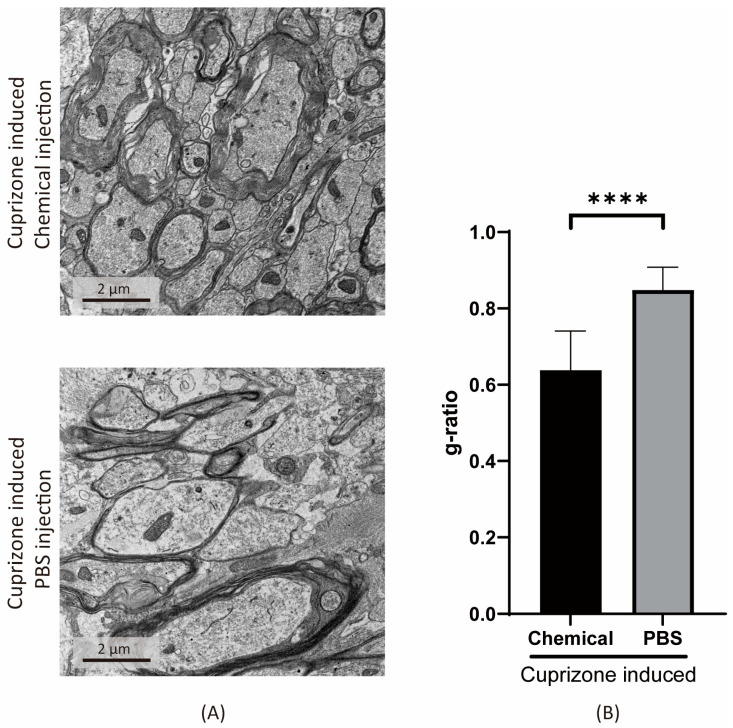
The decrease of g-ratio after chemical cocktail injection into the corpus callosum. B6 mice suffered induced demyelination by consuming a cuprizone diet. The YSF cocktail and PBS were injected directly into the corpus callosum of mice; then, the mice were allowed to recover for two weeks before dissection. Chemical treatment refers to Y27632, SU9516, and forskolin (YSF). (**A**) Electron microscope images of YSF-cocktail- and PBS-injected groups. (**B**) G-ratios are calculated from YSF-cocktail- and PBS-injected groups of mice brain slices. Data are presented as mean ± standard deviation. Significant differences were determined using Student’s *t*-test, * *p* < 0.05, ** *p* < 0.005, *** *p* < 0.001, **** *p* < 0.0001.

We performed electron microscopy (EM) analyses to directly observe the different degrees of remyelination in detail. EM micrographs revealed the thicknesses of myelin sheaths (Figure 7A). To quantify the level of remyelination, a g-ratio was calculated, and a lower g-ratio was observed in the chemical-injected group as compared with the PBS control group (Figure 7B).

## 4. Discussion

We discovered a chemical formulation to convert human fibroblasts into iOLCs within 3 days. These iOLCs displayed OPC-like morphology and expressed the main molecular features of OPCs. Previous studies indicated that the transcription-factor-mediated conversion of mouse fibroblasts into OPCs can be performed by the activity of SRY-Box Transcription Factor 10 (Sox10), Olig2, and zinc finger protein 536 (Zfp536)/NK6 Homeobox 2 (Nkx6.2) in 14 to 21 days [25,26]. In another chemical conversion procedure, Liu et al. published a two-step protocol. At first, they converted mouse fibroblasts into an unstable intermediate cell population of neural lineage cells using M9 medium; then, they redirected the cells by tuning specific culture signals toward the OPC state [30]. The M9 cocktail, including nine small molecules, CHIR99021, LDN193189, A83-01, Hh-Ag1.5, retinoic acid (RA), SMER28, RG108, SB431542, and Parnate, converted mouse fibroblasts into a transition state, and the medium containing LDN193189, SB431542, retinoic acid, and Sonic hedgehog was employed to convert these cells into an OPC-like state in 7 days [30]. The conversion rate was approximately 25% as quantified by Olig2 [30]. In contrast to the induction approach by Liu et al. in mouse cells, our chemical method included three major small molecules, forskolin, Y27632, and SU9516, that converted human cells, which are very different in composition. Our results suggested that the underlying molecular and cellular mechanisms of conversion are likely very different. Our approach generated OPC-like cells more directly and faster than the M9 medium induction method. In our experiments, we treated the cells with VPA, a histone deacetylase inhibitor, for 2 days to eliminate epigenetic signatures and then with Y27632, forskolin, and Su9516 for 1 day for OPC-like cell induction in vitro. Y27632 is a Rho-related kinase (ROCK) inhibitor that promotes stem cell proliferation [45], increases neuroprotection during development [46], and has anti-apoptotic functions [45,47]. Notably, forskolin, a cAMP activator, plays a critical role in the field of the reprogramming of neural lineage cells [48,49,50]. These factors may also support the conversion of fibroblasts into neural cells. In addition, in our cocktail, SU9516, a Cyclin-dependent kinase 2 (Cdk2) inhibitor, played a key role in converting human fibroblasts into OPC-like cells. Previous studies reported that Cdk2 is not essential for mice [51] and the oligodendrocytes in Cdk2 knockout mice have higher differentiation and better remyelination abilities [52]. SU9516 was also reported to improve muscle function by increasing α7β1 integrin of skeletal muscle in a Duchenne muscular dystrophy (DMD) mouse model [53]. This may be related to our findings that inhibition of Cdk2 can convert human fibroblasts into OLCs and promote remyelination in the demyelinated central nervous system.

Although autologous-derived cell transplantation is considered a strategy for the treatment of myelin degeneration, for the practical application of such protocols, there are still many problems to be solved. For example, induced cells are difficult to control due to their heterogeneity, and transplanted cells have low viability after integration. In addition, current viral reprogramming methods for generating iOLCs are inefficient, and only approximately 2–15% of the cells are reprogrammed; however, in the future, an increase in viral titer may increase cell reprogramming efficiency [25,26]. In contrast, chemically generated mouse iOPCs achieve higher reprogramming efficiencies up to a level of approximately 25% Olig2+ and 13% Nkx2.2+ cells [30]. We converted human fibroblasts into iOLCs using a cocktail of small molecules in vitro, measuring 50% or more A2B5+ cells by flow cytometry and achieving a significant increase in reprogramming efficiency. Small molecules enter cells in a short period of time, do not have the problems of insertional mutagenesis caused by viral infections and immune rejection, and are low in cost. Therefore, the rescue of myelin degeneration using a cocktail of small molecules is currently the most economical and convenient cell-free therapeutic method for experimental cell reprogramming in vitro and for developing an in vivo drug therapy.

In previous studies, the majority of mice with myelin degeneration in a cuprizone-induced model was treated with a single agent. These single-treatment modalities have provided insight into the role of a single message on remyelination in vivo. A non-steroidal anti-inflammatory drug (NSAID), indomethacin, is a glycogen synthase kinase 3 beta (GSK3β) inhibitor and promoted both the differentiation of primary oligodendrocytes and remyelination in a 10 week cuprizone-induced-demyelination model [33]. BLZ945, a colony stimulating factor 1 (CSF-1) receptor kinase inhibitor, enhanced remyelination by modulating neuroinflammation in a 5 week cuprizone-induced model [34]. Linagliptin, a dipeptidyl peptidase (DPP)-4 inhibitor used for the treatment of adult type 2 diabetes, was reported to attenuate 4 week cuprizone-induced demyelination by modulating AMP-activated protein kinase/Sirtuin 1 (AMPK/SIRT1) and Janus kinase 2/Signal Transducer And Activator Of Transcription 3/nuclear factor kappa-light-chain-enhancer of activated B cells (JAK2/STAT3/NF-κB) signaling pathways [35]. Ginkgo biloba extract is a traditional Chinese herbal medicine that has neuroprotective, anti-inflammatory, antioxidant, and anti-apoptotic effects [36]. Bilobalide, a component present in Ginkgo biloba extract, inhibited microglia-associated neuroinflammation and promoted remyelination in a cuprizone-induced 6 week model [36]. However, there are two types of cuprizone treatment methods to induce demyelination. The first one is to treat the mice with cuprizone for 12 to 13 weeks to obtain chronically demyelinated lesions [37]. Due to the long-term treatment of cuprizone, the ability for myelin repair endogenously is limited [54,55]. The other model is acute demyelination induced by only feeding the mice with cuprizone for 5–6 weeks. However, if animals are given normal chow after week 5, spontaneous endogenous remyelination occurs. In brief, the short-term cuprizone model may induce acute demyelination, which is more suitable for the study of immunomodulation in MS. Therefore, acute cuprizone-induced demyelination should not be used to study the effects of factors on remyelination. To study remyelination in a non-supportive environment, a chronic cuprizone model must be selected [37,55,56]. Therefore, to avoid the effects of autologous myelin repair on the experimental results, we treated mice with cuprizone for 12 weeks to obtain chronic, long-term demyelinating lesions and found that our small molecule formulation effectively rescued the cuprizone-induced degeneration of myelin in the mouse corpus callosum.

Although there may have been some glial activation in both the cuprizone-treated PBS and 3C-cocktail-treated groups, the 3C-cocktail-treated group displayed oligodendrocyte recovery at a level significantly higher than the control as shown by Luxol fast blue staining (Figure 6A), Olig2 (Figure S1), PLP (Figure 6B), and MBP (Figure 6B).

## 5. Conclusions

We report that a small-molecule cocktail has the potential to reprogram human fibroblasts into an oligodendrocyte-like state based on displaying a similar morphology and specific oligodendrocyte markers. Furthermore, injection of the chemical cocktail promoted the process of remyelination in the corpus callosum of cuprizone-induced demyelinated mice. Combined, our results provide a proof-of-principle that a chemical cocktail can generate oligodendrocyte-like cells and rescue demyelination in vivo. Our research provides a starting point for the development of a procedure to use regenerative medicine as a potential cell-free therapy for MS treatment in the future.

## 6. Patents

“Methods for producing oligodendrocyte lineage cells by reprogramming somatic cells with small molecules” has been applied for as a patent. “Methods of treating demyelinating diseases” will be filed as a patent application.

## Data Availability

Western blot original files are provided.

## Supplementary Materials

The following supporting information can be downloaded at: https://www.mdpi.com/article/10.3390/cells11071091/s1, Table S1: Cell culture reagents used in this study; Table S2: Chemical compounds used to induce OLCs from fibroblasts; Table S3: Antibody list for immunofluorescence, Western blotting, and flow cytometry; Table S4: List of primers used in qRT-PCR from 5′ to 3′; Figure S1: Three-chemical (3C)-cocktail injection restored Olig2 expression in mice.
