# Supplementary material for: Development of a Chemical Cocktail That Rescues Mouse Brain Demyelination in a Cuprizone-Induced Model
Source: Cells. 2022 Mar 24;11(7):1091. doi: 10.3390/cells11071091 (PMC8997971; doi:10.3390/cells11071091)
Supplement: Supplementary file 1 [file cells-11-01091-s001.zip › cells-1625799-supplementary.pdf]

Table S1: Cell culture reagents used in this study

Chemicals & Media	Company	Catalogue
Y-27632 (ROCKi)	LC Laboratories	Y-5301
SU9516 (Cdki)	R&D Systems	2907
Forskolin (FSK)	Tocris	1099
Valproic acid (VPA)	Tocris	2815
bFGF	Pepro Tech	100-18B
EGF	Pepro Tech	AF-100-15
PDGFAA	Pepro Tech	100-13A
NT3	Pepro Tech	450-03
DMEM	Life Technologies	11965-084
DMEM/F12	Life Technologies	11320-033
N2 supplement	Life Technologies	17502-048
B27 supplement	Life Technologies	17504-044
Accutase	Innovative Cell	AT-104
Trypsin	Life Technologies	15400-054

Table S2: Chemical compounds used to induce OLCs from fibroblasts

Name	Mechanism	Source	Concentration	Structure
Valproic acid	Histone deacetylase inhibitor	TOCRIS	3 mM	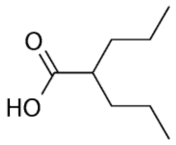
Y-27632	ROCK inhibitor	LC Laboratories	10 μM	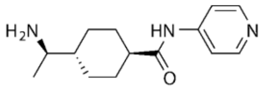
Forskolin	cAMP activator	TOCRIS	10 μM	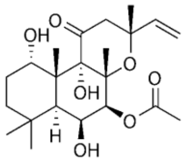
SU9516	CDK inhibitor	R&D Systems	10 μM	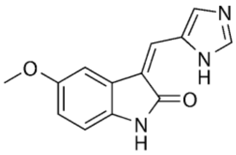

Table S3: Antibody list for immunofluorescence, western blotting and flow cytometry

Antibody	Company	Catalogue
Olig2-antibody	Millipore	AB9610
O4-antibody	Millipore	MAB345
MBP-antibody	Proteintech	10458-1-AP
PDGFRa-antibody	R&D Systems	MAB322
GalC-antibody	Proteintech	11991-1-AP
GPR17-antibody	Proteintech	13416-1-AP
O1-antibody	Millipore	MAB344
Nkx2.2-antibody	Proteintech	13013-1-AP
Myelin PLP-antibody	Abcam	AB28486
DAPI	Life Technologies	D1306
Goat anti-mouse IgM CF555	Life Technologies	A-21426
Goat anti-mouse IgG CF488	Biotium	20018
Goat anti-mouse IgG CF555	Life Technologies	A21424
Goat anti-rat IgG CF555	Life Technologies	A21434
Goat anti-rabbit IgG CF488	Biotium	20019
Goat anti-rabbit IgG CF555	Life Technologies	A21429
Anti-A2B5-APC	Miltenyi Biotec	130-093-582

Table S4: List of primers used in qRT-PCR written 5′-to-3′

Primer	Sequence
MBP-F	TGG TGA TGG AGA TGT CAA GCA GGT
MBP-R	GCT GTG GTT TGG AAA CGA GGT TGT
PLP-F	CCA GAG GCC AAC ATC AAG CT
PLP-R	TCG GGA TGT CCT AGC CAT TT
GAPDH-F	CCA CTC CTC CAC CTT TGA CG
GAPDH-R	CCA CCA CCC TGT TGC TGT AG

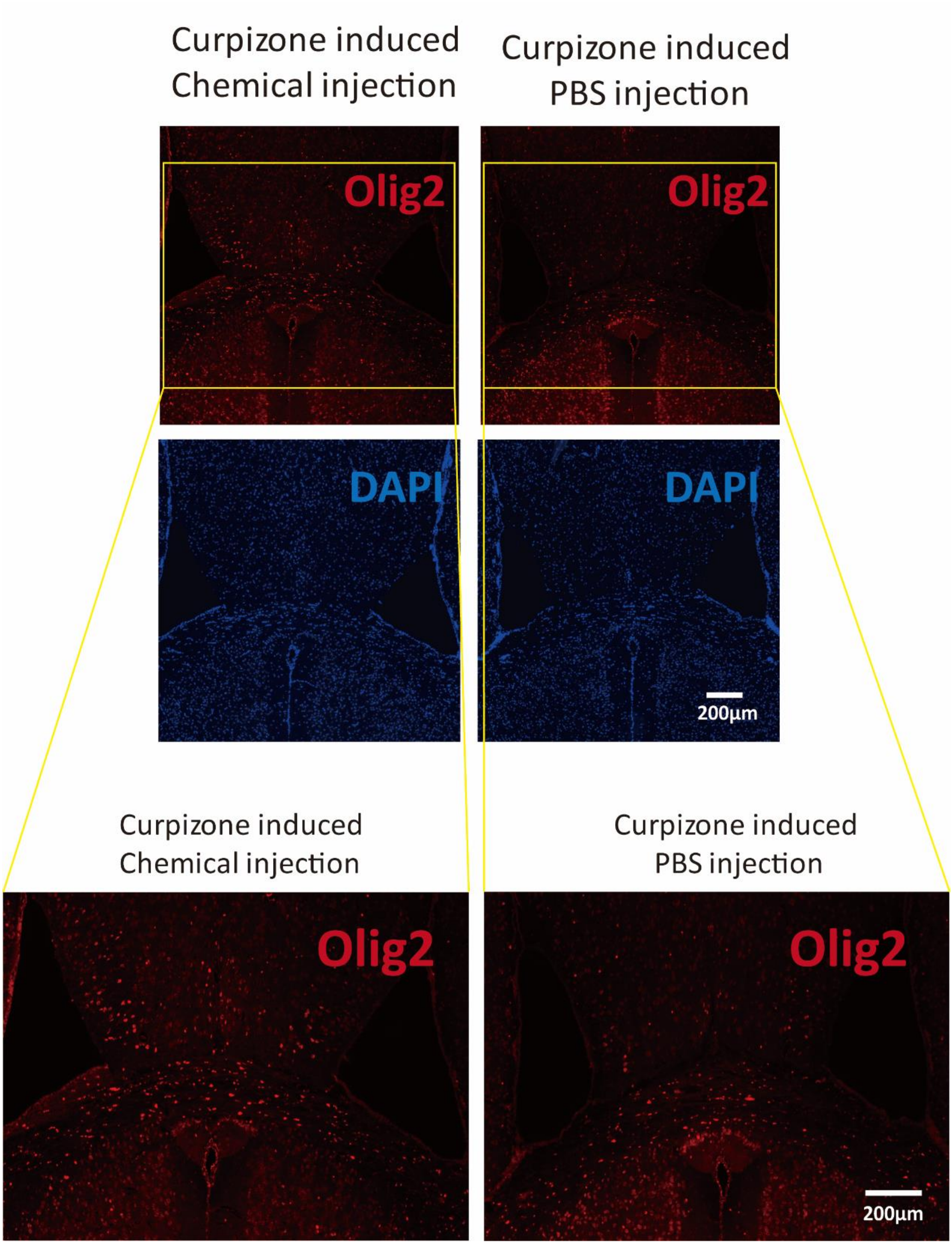

Figure S1. 3 chemical (3C) cocktail injection restored Olig2 expression in mice. Induction of demyelination in B6 mice by cuprizone. The YSF-cocktail or PBS was injected directly into the corpus callosum of mice. Then the mice were allowed to recover for two weeks before dissection. YSF refers to Y27632, SU9516 and forskolin. After 2 weeks of recovery, immunofluorescence staining was performed to observe the Olig2 (red) protein expression of the YSF-cocktail and PBS-injected groups. DAPI (blue) was used to stain nuclei.
